# Structure and Microbiological Activity of 1*H*-benzo[*d*]imidazole Derivatives

**DOI:** 10.3390/ijms24043319

**Published:** 2023-02-07

**Authors:** Andrzej Olczak, Tomasz Pawlak, Sylwia Kałużyńska, Katarzyna Gobis, Izabela Korona-Głowniak, Katarzyna Suśniak, Marcin Zaborowski, Małgorzata Szczesio

**Affiliations:** 1 Institute of General and Ecological Chemistry, Faculty of Chemistry, Lodz University of Technology, Zeromskiego 116, 90-924 Lodz, Poland; 2 Centre of Molecular and Macromolecular Studies, Polish Academy of Science, Sienkiewicza 112, 90-363 Lodz, Poland; 3 Department of Organic Chemistry, Faculty of Pharmacy, Medical University of Gdańsk, Gen. Hallera Ave. 107, 80-416 Gdańsk, Poland; 4 Department of Pharmaceutical Microbiology, Faculty of Pharmacy, Medical University of Lublin, Chodźki Street 1, 20-093 Lublin, Poland; 5 Dean Office’s, Faculty of Chemistry, Lodz University of Technology, Zeromskiego 114, 90-543 Lodz, Poland

**Keywords:** 1*H*-benzo[*d*]imidazole derivatives, antimicrobial activity, structure–activity relationship, X-ray, ADME, crossing the blood–brain barrier

## Abstract

Three new crystal structures of 1*H*-benzo[*d*]imidazole derivatives were determined. In the structures of these compounds, an identical system of hydrogen bonds, C(4), was observed. Solid-state NMR was applied for testing the quality of the obtained samples. All of these compounds were tested for in vitro antibacterial activity against Gram-positive bacteria and Gram-negative bacteria, as well as antifungal activity, by checking their selectivity. ADME calculations indicate that the compounds can be tested as potential drugs.

## 1. Introduction

Many common infections today, such as pneumonia and urinary tract infection, do not respond to standard treatment. This extends treatment time and causes higher mortality. In the United States, drug-resistant bacteria cause over 2.8 million infections and approximately 35,000 deaths annually [1,2]. In Europe, AMR (antimicrobial resistance) causes approximately 33,000 deaths annually [3,4]. It is estimated that, in 2050, people will die more often from infections caused by antibiotic-resistant strains than from cancer [5]. The problem of drug resistance has been noticed by the CDC (Centers for Disease Control and Prevention) and the WHO (World Health Organization), as well as European and US governments. If coordinated global action is not taken immediately, we may face serious medical, social and economic complications in just a few years [1,2,4,5,6].

The extensive use, or even abuse, of antimicrobial drugs in medicine, animal husbandry and agriculture has resulted in strong selection pressure for the emergence and spread of various resistance mechanisms among bacteria [7]. According to a WHO report from 2022, the global problem of drug resistance is caused by bacteria that can cause both hospital and community-acquired infections. The most important ones among them are *Escherichia coli* (urinary tract infections, bacteremia/sepsis, nosocomial pneumonia and others)—resistant to third-generation cephalosporins and fluoroquinolones; *Klebsiella pneumoniae* (urinary tract infections, pneumonia, bacteraemia/sepsis and others)—resistant to third-generation cephalosporins and carbapenems; *Staphylococcus aureus* (bacteremia/sepsis, skin and soft tissue infections, osteoarthritis and others)—methicillin-resistant (MRSA) bacteria, resistant to all β-lactams except for ceftaroline and ceftobiprole); *Streptococcus pneumoniae* (pneumonia, bacteremia/sepsis, meningitis and otitis media)—insensitive or resistant to penicillin; *Salmonella* (zoonotic salmonellosis)—resistant to fluoroquinolones; and *Shigella* (bacterial dysentery)—resistant to fluoroquinolones [6].

Drug-resistant strains of fungi, increasingly isolated in hospital environments, are also a problem. The fungus *Candida albicans* is most often responsible for infection, but more and more infections caused by other fungi are starting to appear [8]. It should be emphasized that, in recent years, the number of cases of fungemia caused by species other than *C. albicans* (*C. glabrata*, *C. krusei*, *C. parapsilosis*, *C. lusitaniae* and *C. tropicalis*) has increased by almost 2.5 times, which accounts for approx. 40% of fungi isolated from blood in ICUs (intensive care units) and up to 70% in hematology wards [9]. Unfortunately, the overuse of antifungal drugs, especially from the azole group, leads to the natural selection of resistant strains. Due to the fact that we do not have a very wide arsenal of agents to fight infections, their use should be thoughtful and prudent [10].

In the face of the spread of MDR (multidrug-resistant), XDR (extensively drug-resistant—sensitive to no more than two groups of antibiotics) and PDR (pandrug-resistant—resistant to all groups of antibiotics) microorganisms, it is necessary to implement a global strategy to prevent microbial drug resistance, mainly consisting in reducing the use of antimicrobial drugs; increasing the number of effective antimicrobial drugs active against resistant microbes, including the establishment of a global fund to support innovation in research on new drugs in early phases and non-commercial ones; promoting investment in new drugs and improving existing ones; and building a global coalition for action against drug resistance [4,5,6].

**Figure 1:**
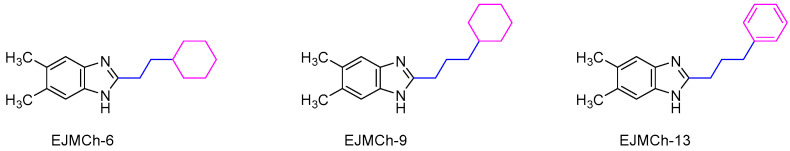
Structural formulas of the tested compounds.

Therefore, we have undertaken the search for new compounds active against microorganisms resistant to antimicrobial drugs. Our interests include benzimidazoles, which exhibit multidirectional biological activity [11]. It was found, among others, that antibacterial and antifungal agents can be found in this group [12,13]. We focused in particular on benzimidazoles substituted at the C-2 position with cyclohexylethyl, cyclohexylpropyl and phenylpropyl moieties (Figure 1). These compounds were tested for tuberculostatic activity against *M. tuberculosis* [14]. These studies were conducted because drug-resistant and multidrug-resistant strains of *M. tuberculosis* have appeared [15,16]. The antitubercular activity of the **EJMCh-6** compound is very high, unlike that of others presented in this work. Additionally, this compound is not toxic to eukaryotic cells [14].

**Figure 2:**
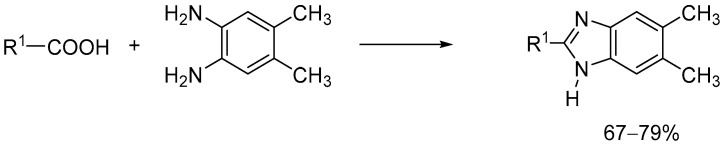
Synthesis of desired compounds.

The paper presents new crystal structures of three benzimidazoles substituted at the C-2 position with cyclohexylethyl, cyclohexylpropyl and phenylpropyl moieties (Figure 2). All of these compounds were tested for in vitro antibacterial activity against Gram-positive bacteria and Gram-negative bacteria, as well as antifungal activity, by checking their selectivity.

## 2. Results and Discussion

### 2.1. Structure Analysis

**Figure 3:**
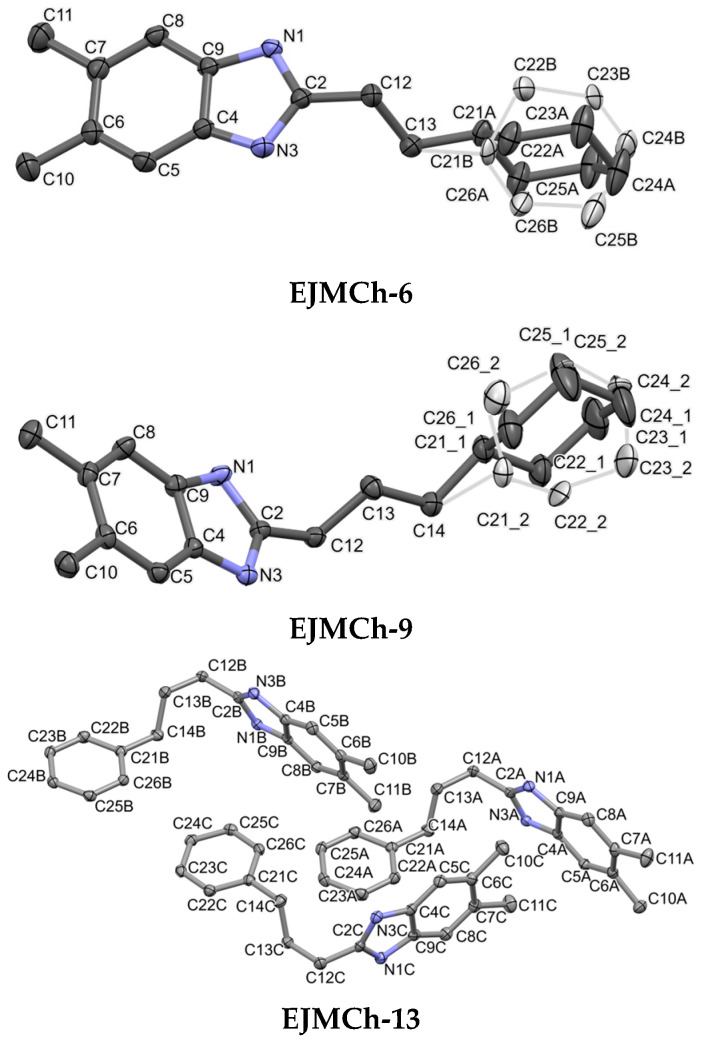
Molecular structures of all compounds showing atom-labeling schemes. Displacement ellipsoids are drawn at the 50% probability level. Drawings were prepared with Mercury software 2022.3.0 [18]. Light gray color (**EJMCh-6** and **EJMCh-9**) shows alternative conformations of disordered rings.

Crystallographic data are included in Table 1, and the ORTEP representation of the structures is presented in Figure 3. Both **EJMCh-6** and **EJMCh-9** structures contain one molecule per asymmetric unit. Interestingly, they both show similar disorder in the cyclohexane ring (Figure 3). The ratios of the refined components are 0.79:0.21 and 0.84:0.16 for **EJMCh-6** and **EJMCh-9**, respectively. In contrast, the **EJMCh-13** compound contains three independent molecules in the asymmetric unit.

**Figure 4:**
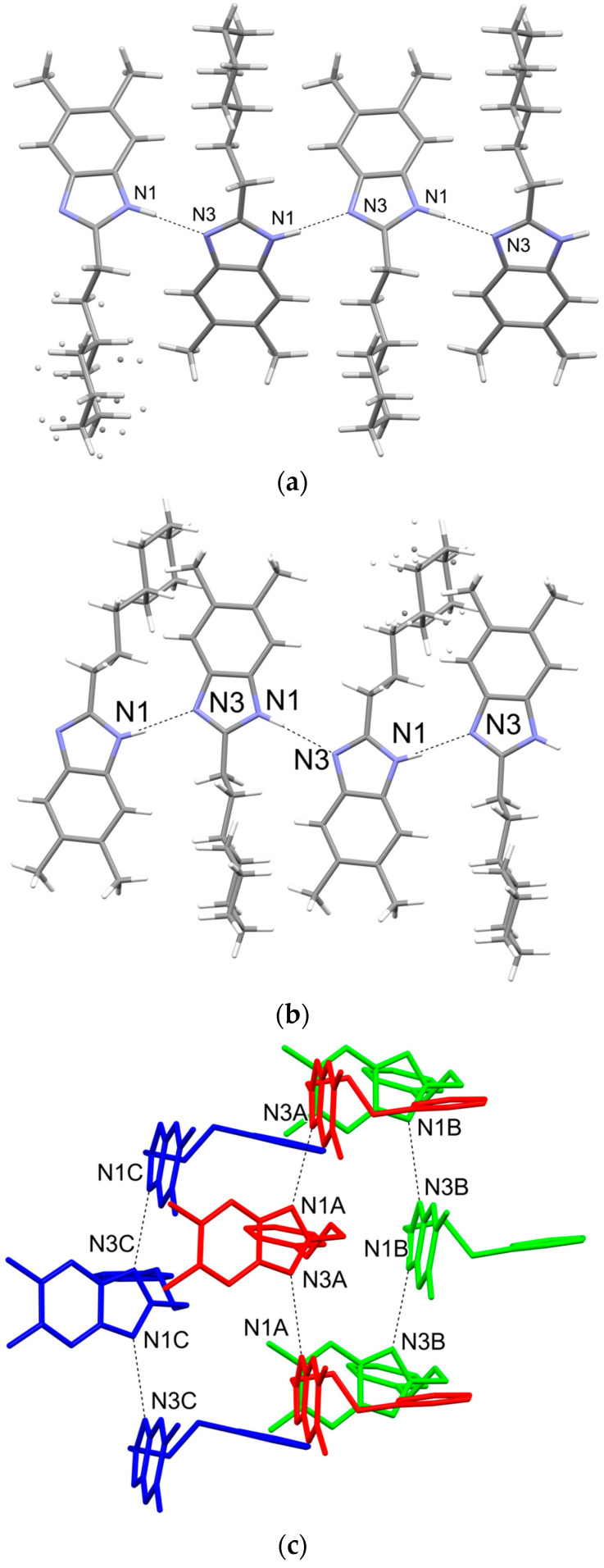
(**a**) Intermolecular hydrogen bonds in **EJMCh-6**. (**b**) Intermolecular hydrogen bonds in **EJMCh-9** (**c**) Intermolecular hydrogen bonds in **EJMCh-13**. The colors indicate three different molecules in the asymmetric unit and their crystallographic symmetry equivalents.

In all studied structures, the same pattern of hydrogen bonds of type N1-H1…N3 is adopted (Figure 4). The system of hydrogen bonds observed in all structures is described as a chain, C(4), according to hydrogen-bond graph–set theory [19]. Interestingly, each of the independent molecules only forms a chain with its symmetrical counterparts, and all three chains align along the [001] crystallographic direction.

**Figure 5:**
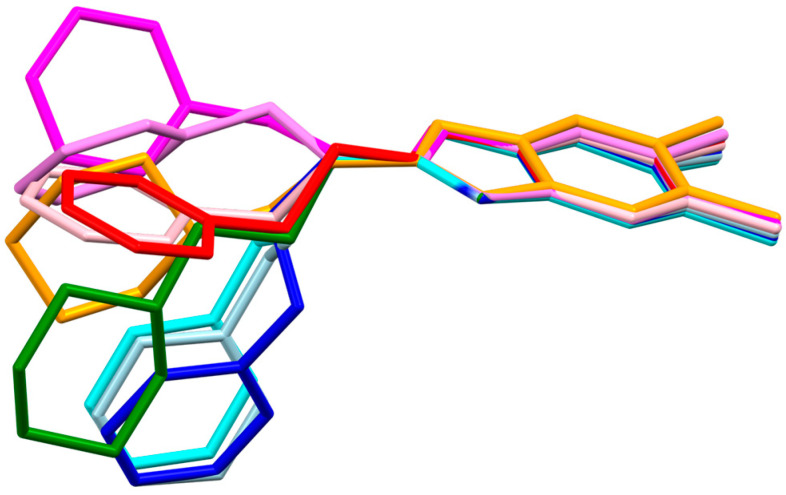
Superimposition of the studied molecules and HIQXIR (HIQXIR—red, pink, magenta and violet; **EJMCh-9**—green; **EJMCh-6**—orange; **EJMCh-13**—light blue, blue and cyan).

In the CSD (Cambridge Structural Database) [20], there is only one analogous structure, with code HIQXIR [21]. It has a phenyl end group and an ethyl linker (CH_2_-CH_2_). This structure, unlike the other three, is a hydrate in the crystal state. All the above-mentioned compounds with an ethyl linker have a stretched form (Figure 5), while the longer, three-carbon ones have a bent form. This is one of the factors that may affect the antibacterial activity of the tested compounds. A second factor affecting the activity may be the presence of an aromatic ring.

#### Sample Quality Verification via Solid-State NMR

**Figure 6:**
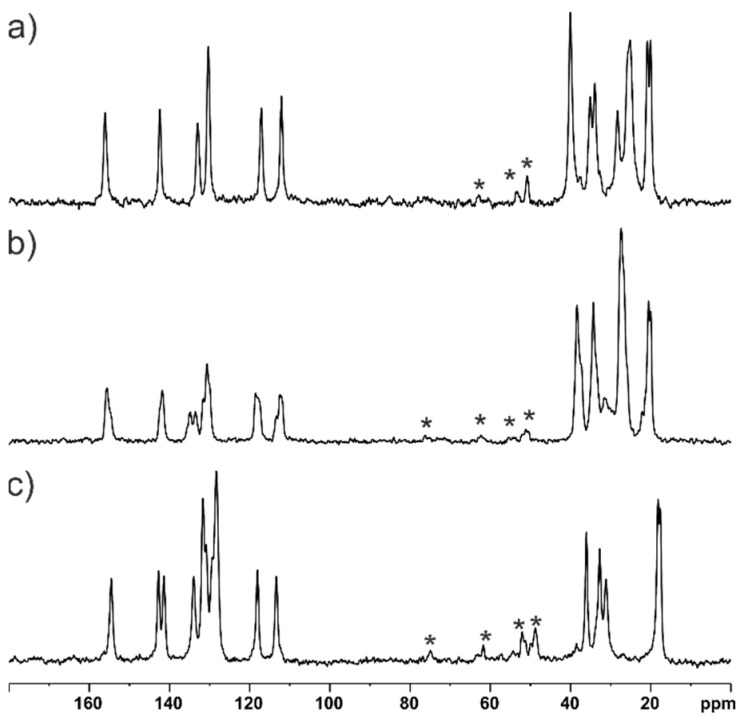
^13^C CP MAS spectra of **EJMCh-6** (**a**), **EJMCh-9** (**b**) and **EJMCh-13** (**c**) recorded at a spinning rate of 8 kHz. The recycle delay was 3 s. Asterisks indicate spinning sidebands.

The quality of the samples was tested by employing the solid-state NMR technique. Figure 6 shows the ^13^C CP MAS NMR spectra of all studied samples. From the analysis of the experimental data, it is clear that all of them represent well-crystalline structures. Counting the number of isotropic resonance lines in the ^13^C CP MAS NMR spectra of **EJMCh-6** and **EJMCh-9,** it is apparent that the number of molecules in the asymmetric unit is the same as that determined in the X-ray study. **EJMCh-13** should have a visible triplication of signals, at least for some positions. However, this is not the case, likely due to the fact that all three molecules in the asymmetric unit cell have very similar conformation and interatomic distances, resulting in no visible triplication of signals. Another reason could be the high broadening of the signals.

### 2.2. ADME Analysis

**Figure 7:**
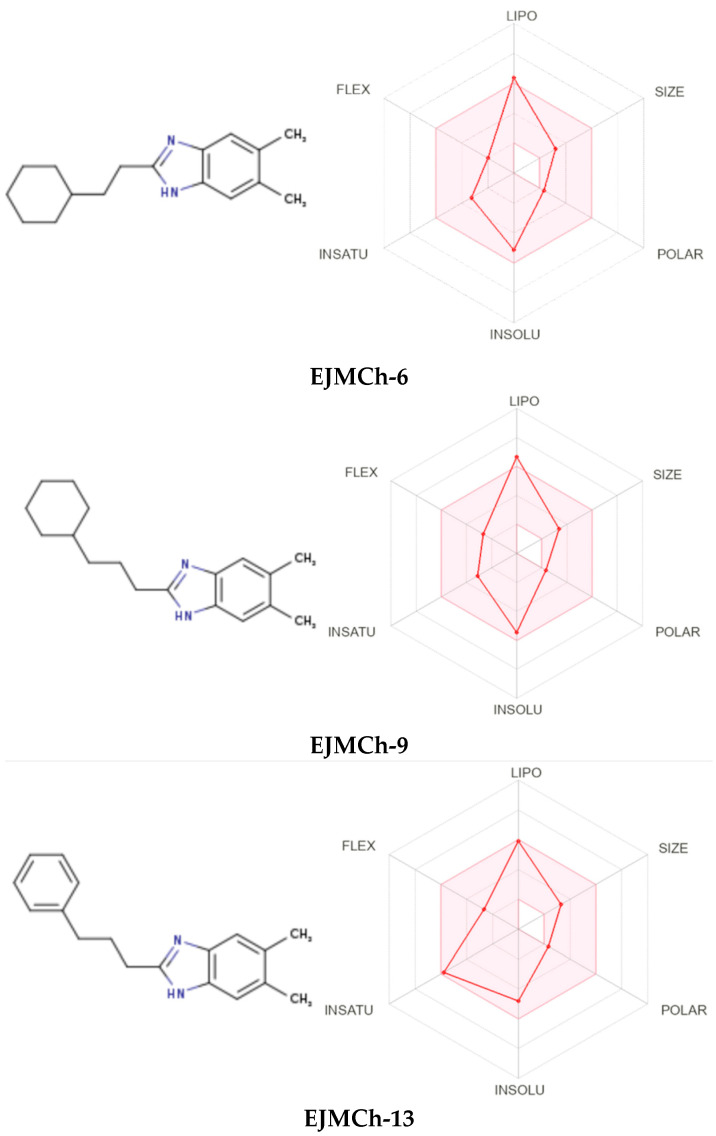
Bioavailability radars of all compounds.

Bioavailability radars for all studied compounds were made (Figure 7). For drug-like properties, the compounds were found to have a good bioavailability score (0.55). Six physicochemical properties are considered on the bioavailability radar: lipophilicity, size, polarity, solubility, flexibility and saturation. Compound **EJMCh-13**, which contains the phenyl group instead of cyclohexane, has a better lipophilicity index, while the saturation index (fraction of carbons in the sp3 hybridization not less than 0.25) remains within a satisfactory range. All the compounds conform to the rules of Lipinski [22], Ghose [23], Egan [24], Veber [25] and Muegge [26] and thus are good drug candidates. The logKp values of the tested compounds range from −4.48 cm/s to −3.58 cm/s, and the more negative logKp is, the less the molecule penetrates the skin.

**Figure 8:**
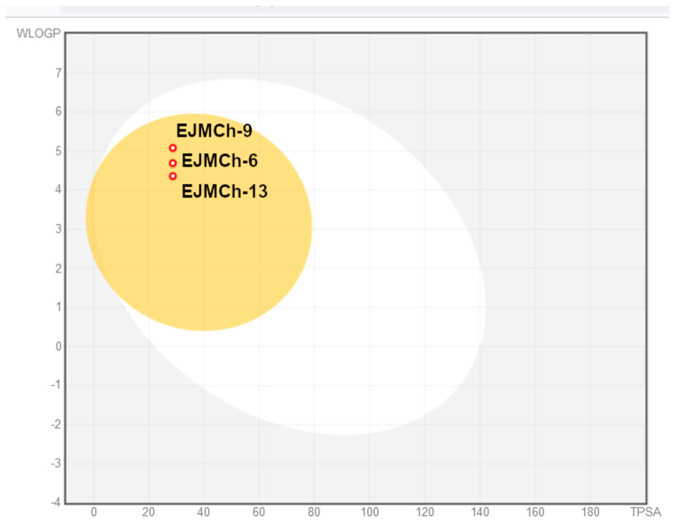
BOILED-Egg diagram for all compounds (lipophilicity (WLOGP) and polarity (tPSA)). Human intestinal absorption—white area; blood–brain barrier permeation—yellow area.

The BOILED-Egg diagram (brain or intestinal estimated permeation predictive model) indicates that all compounds can be absorbed in the gastrointestinal tract, which may make them effective drugs (Figure 8). The analysis confirmed the effectiveness of the tested compounds in crossing the blood–brain barrier. This may be of particular importance in the treatment of tuberculous meningitis, which is the most severe and life-threatening form of tuberculosis. Despite the cure, more than half of patients suffer from permanent damage to the nervous system [27].

### 2.3. Antimicrobial Activity

The increase in and spread of antimicrobial resistance to available drugs necessitate a search for new molecules for the treatment of bacterial infections. Benzimidazole and its derivatives are the most potent classes of molecules against microorganisms. They were found as heterocyclic aromatic compounds with a variety of biological activities, such as anti-inflammatory, antiparasitic, antimalarial, antimycobacterial and antiviral activities, which was nicely reviewed by Tahlan et al. [28]. There are a number of derivatives evaluated for their antimicrobial activity against selected microbial species: 2-substituted-1H-benzimidazole derivatives, 2-mercaptobenzimidazole derivatives, 2-mercaptobenzimidazole and β-lactum segment derivatives containing –CONH–, new benzimidazoles bearing 2-pyridone, 3,4-dihydro triazino[1,2-a]benzimidazole compounds and novel substituted benzimidazole carboxamidine [28]. Most of these novel derivatives revealed good antimicrobial activity, ranging from MIC = 0.39–0.78 mg/L in the new class of benzimidazole and phenyl-substituted benzyl ethers or 0.12–0.5 mg/L in new bis-benzimidazole diamidine compounds to MIC = 100–250 mg/L in novel series of pyrido[1,2-a]benzimidazole derivatives [28].

The results of the antibacterial and antifungal activities of benzimidazoles substituted at the C-2 position with cyclohexylethyl, cyclohexylpropyl and phenylpropyl moieties tested in this study are presented in Table 2 as MICs, i.e., the lowest concentrations of compounds that prevent visible growth of the organism, and MBCs, i.e., the lowest concentrations that result in a ≥99.9% reduction in the microorganism inocula upon subculture with compound-free medium. Vancomycin, ciprofloxacin and nystatin were used as the standard drugs. **EJMCh-6** was found to be without bioactivity against the tested reference strains. Noticeably, the tested **EJMCh-13** and **EJMCh-9** showed no bioactivity against Gram-negative bacteria (*E. coli* ATCC 25922, *S.* Typhimurium ATCC 14028, *K. pneumoniae* ATCC 13883 and *P. aeruginosa* ATCC 9027; MIC >1000 mg/L) or mild bioactivity (*P. mirabilis* ATCC 12453) at minimal inhibition concentration (MIC = 250). The MIC values for Gram-positive reference bacteria indicate strong (MIC 15.6 mg/L) anti-staphylococcal (*S. aureus* ATCC 25923 and *S. epidermidis* ATCC 12228) and very strong anti–micrococcal (*M. luteus* ATCC 10240) activities of the **EJMCh-13** compound. The tested **EJMCh-9** and **-13** compounds also showed strong activity against enterococci and spore-forming pathogen *Bacillus cereus*. **EJMCh-9** presented good bioactivity against *S. aureus* strains (including MRSA) and very strong activity against reference *S. epidermidis* and *M. luteus* strains. The high values of the MBC/MIC ratio (8–16) for **EJMCh-9** and **EJMCh-13** indicate their bacteriostatic activities, except for the bactericidal activity of **EJMCh-13** against *S. epidermidis* and *B. cereus* reference strains (MBC/MIC 1). Surprisingly, the antifungal bioactivity (*C. albicans* ATCC 2091, *C. glabrata* ATCC 90030 and *C. parapsilosis* ATCC 22019) of the tested compounds was not observed.

The mechanism of action of the tested derivatives against Gram-positive and Gram-negative bacteria is not yet clearly understood. The biological activity against these two groups of bacteria could be different due to their cell wall structure differences and thus the difference in permeability. Peptidoglycan is a major component (90%) of the Gram-positive cell wall, whereas in Gram-negative bacteria, peptidoglycan, constituting 10% of the cell wall, lies between the cytoplasmic membrane and the outer lipid bilayer containing lipopolysaccharide, porins and adhesins, which create an additional barrier to overcome. The yeast cell wall is a characteristic structure of fungi and is mainly composed of glucans, chitin and glycoproteins, forming a different type of barrier. The dissimilar activity against Gram-positive bacteria of **EJMCh-9** and **EJMCh-13** may result from their different lipophilicity, stacking interactions and the possibility of hydrogen bonding.

## 3. Materials and Methods

### 3.1. Chemistry

The compounds were synthesized using simple synthesis from an appropriate diamine and carboxylic acid according to the methods previously described: Method A (**EJMCh-6**): carboxylic acid (1.5 equiv.), 5,6-dimethy-1,2-diaminobenzene (1 equiv.), and PPA (polyphosphoric acid) at 180–200 °C; NaHCO_3_/H_2_O. Method B (**EJMCh-9** and **EJMCh-13**): carboxylic acid (1.5 equiv.) and 5,6-dimethyl-1,2-dimanobenzene (1 equiv.) at 160–180 °C; NaOH/H_2_O (Figure 2) [14]. The IR spectra are shown in Supporting Information: Figure S1 (**EJMCh-6**), Figure S2 (**EJMCh-9**), Figure S3 (**EJMCh-13**).

### 3.2. X-ray Study

Single crystals of compounds suitable for X-ray diffraction were obtained with the slow evaporation of solvents at room temperature from methanol–DMF (1:1 *v*/*v*) solutions. The diffraction measurements were performed with Bruker SMART APEXII CCD Diffractometer (Bruker AXS Inc., Madison, WI, USA) at low temperature (100 K) and under CuK_α_ (1.54184 Å) radiation. The diffraction data were processed with SAINT ver. 8.34A, XPREP ver. 2014/2 and SADABS ver. 2014/4 for structures **EJMCh-6** and **EJMCh-13**, and TWINABS ver. 2008/4 for structure **EJMCh-9** (Bruker AXS Inc., Madison, WI, USA). Crystal structure solution and refinement were carried out with SHELX [29,30] and SHELXLE for visualization [31]. All H atoms (except for those engaged in hydrogen bonds) were geometrically optimized and considered riding atoms, with distances appropriate for 100 K temperatures and with U_iso_(H) = 1.2 U_eq_(C, N). The methyl H atoms were refined with U_iso_(H) = 1.5 U_eq_(C). CCDC 2231627, 2231628 and 2231629 contain the supplementary crystallographic data for this paper. The data are provided free of charge by The Cambridge Crystallographic Data Center at www.ccdc.cam.ac.uk/structures (accessed on 13 December 2022). The graphics of the crystal structures were obtained with Mercury [18]. PublCIF was used in data preparation [32]. In the aliphatic rings, structures **EJMCh-6** and **EJMCh-9** displayed some disorder, which was resolved with DSR [33].

### 3.3. NMR Study

^13^C cross-polarization magic-angle spinning (CP MAS) NMR experiments were carried out with an Avance III spectrometer equipped with HX MAS probe heads of 4 mm, operating at 400.19 and 100.63 for ^1^H and ^13^C, respectively. The MAS frequency was 8 kHz. The CP MAS experiments were performed with the proton 90° pulse length of 3.4 μs and a contact time of 2 ms. For cross-polarization, the nutation frequency was 63 kHz with a ^1^H ramp shape from 90% to 100%. A sample of U-^13^C, ^15^N-labeled histidine hydrochloride was used to set the Hartmann–Hahn condition. A total of 3.5k data points were acquired for the spectral width of 50 kHz. There were no special preparations of samples prior to the solid-state NMR study. The ^1^H and ^13^C NMR liquid-state spectra of all samples are shown in supplementary material information. All the spectra were recorded in DMSO-d_6_ with a Neo Bruker 400 MHz spectrometer and are consistent with the results provided in [14], where we investigated the same set of samples. **EJMCh-6** ^1^H NMR (Figure S4): δ 0.86–0.94 (m, 2H, CH_2_), 1.10–1.23 (m, 4H, 2CH_2_), 1.60–1.75 (m, 6H 3CH_2_ and 1H CH), 2.27 (s, 6H, 2CH_3_), 2.75 (t, 2H, CH_2_), 7.14 (s, 1H, ArH), 2.25 (s, 1H, ArH), 11.8 (s, 1H, NH); ^13^C NMR (Figure S5): δ 20.4, 26.2, 26.5, 26.6, 33.0. 35.7, 37.1, 111.3, 118.7, 129.1, 129.9, 133.2, 142.4, 154.8. **EJMCh-9** ^1^H NMR (Figure S6): δ 0.81 (br m, 2H, CH_2_), 1.17 (br m, 6H, 3CH_2_), 1.63–1.70 (br m, 7H, 3CH_2_ and 1H CH), 2.24 (s, 6H, 2CH_3_), 2.68 (br m, 2H, CH_2_), 3.33 (br m, 2H, CH_2_), 7.12 (s, 1H, ArH), 7.23 (s, 1H, ArH), 11.84 (s, 1H, NH); ^13^C NMR (Figure S7): δ 21.9, 27.1, 27.9, 28.2, 30.9, 34.8, 38.6, 38.8, 112.8, 120.3, 130.6, 131.5, 134.8, 144.0, 156.2. **EJMCh-13** ^1^H NMR (Figure S8): δ 2.04 (m, 2H, CH_2_), 2.27 (s, 6H, 2CH_3_), 2.64 (t, 2H, CH_2_), 2.76 (t, 2H, CH_2_), 7.15–7.17 (m, 2H, ArH), 7.19–7.23 (m, 3H, ArH), 7.30 (s, 2H, ArH), 11.90 (s, 1H, NH); ^13^C NMR (Figure S9): δ 20.4, 28.5, 29.8, 35.1, 111.3, 118.8, 126.3, 128.8, 128.9, 129.2, 130.1, 133.2, 142.1, 142,4, 154.2.

### 3.4. ADME

The compounds were analyzed for their pharmacokinetic properties, drug likeness and absorption. ADME (absorption, distribution, metabolism and excretion) analysis was performed using the SwissADME service 2022 [34,35] and BOILED-Egg—to predict the gastrointestinal absorption and brain penetration of molecules [36].

### 3.5. In Vitro Antibacterial Activity Assay

All target compounds were screened for antibacterial and antifungal activities using the 2-fold micro-dilution broth method. The minimal inhibitory concentration (MIC) of the tested compounds was evaluated for a panel of reference Gram-positive bacteria (*Staphylococcus aureus* ATCC 25923, *S. aureus* ATCC BAA-1707, *Staphylococcus epidermidis* ATCC 12228, *Micrococcus luteus* ATCC 10240 and *Bacillus cereus* ATCC 10876) and Gram-negative bacteria (*Salmonella* Typhimurium ATCC 14028, *Escherichia coli* ATCC 25922, *Proteus mirabilis* ATCC 12453, *Klebsiella pneumoniae* ATCC 13883, *Pseudomonas aeruginosa* ATCC 9027 and yeasts: *Candida albicans* ATCC 102231, *Candida parapsilosis* ATCC 22019 and *Candida glabrata* ATCC 90030). The procedure for conducting antimicrobial activity testing was previously described in detail [37]. The solutions of the tested compounds in dimethylosulfoxide (DMSO) were suspended in Mueller–Hinton broth for bacteria or Mueller–Hinton broth with 2% glucose for fungi. Then, series of two-fold dilutions were carried out in sterile 96-well polystyrene microtitrate plates (Nunc, Roskilde, Denmark), obtaining concentrations from 1000 to 7.8 µg/mL in the appropriate medium. Simultaneously, the inocula of 24 h cultures of microorganisms in sterile physiological saline (0.5 McFarland standard density) were prepared and added to each well, obtaining the final density of 1.5 × 105 CFU/mL for bacteria and 5 × 10^4^ CFU/mL for yeasts; CFU—colony forming units. Proper positive (inoculum without tested compound) and negative (compound without inoculum) controls were added to each microplate. Vancomycin, ciprofloxacin and nystatin were used as the standard reference reagents.

After incubation (35 °C, 24 h) the growth of microorganisms was measured spectrophotometrically at 600 nm. MICs were marked at the lowest dilution of 1H-benzo[d]imidazole derivatives without the growth of bacteria or yeasts.

Then, 5 µL of the suspension from each well, including controls, was subcultured on the agar plates in order to determine the minimal bactericidal concentration (MBC) or minimal fungicidal concentration (MFC). The plates were incubated at 35 °C for 24 h. The MBC/MFC was determined at the lowest concentration of derivatives inhibiting the growth of microbes. The MBC/MIC index was also calculated to show the bacteriostatic or bactericidal effect of the tested compounds.

## 4. Conclusions

Studies on the selectivity of the biological activity of compounds with known antitubercular activity were carried out. The tuberculostatic activity of **EJMCh-6** is very high, unlike that of other compounds presented in this work. On the other hand, in **EJMCh-9** and **EJMCh-13**, significant activity against other Gram-positive bacteria can be observed. The presence of an odd propyl linker may cause an increase in activity against other bacteria. The molecules of this compound are bent, unlike the molecules with an ethyl linker, which are more stretched. It seems that the decisive factor regarding the biological activity of the tested compounds is the length of the linker. Compounds with a longer linker have more conformational freedom, which can make them more flexible to fit the binding site. Despite the conformational differences, compounds with propyl and ethyl linkers form the same arrangement of hydrogen-bond chains in the crystal. The in silico ADME analysis shows that all three tested 1H-benzo[*d*]imidazole derivatives are good candidates as potential drugs.

## Figures and Tables

**Table 1:** Crystal data, data collection and refinement details of all compounds.

	EJMCh-6	EJMCh-9	EJMCh-13
Crystal data			
Chemical formula	C_17_H_24_N_2_	C_18_H_26_N_2_	C_18_H_20_N_2_
*M*_r_	256.38	270.41	264.36
Crystal system, space group	Monoclinic, *Cc*	Monoclinic, *P*2_1_/*c*	Monoclinic, *Cc*
*a*, *b*, *c* (Å)	15.2276 (3), 12.7173 (3), 9.7501 (2)	14.2078 (4), 9.9210 (3), 11.5761 (4)	28.8210 (9), 15.4123 (5), 10.1758 (3)
β (°)	126.1959 (5)	104.7986 (9)	98.2484 (13)
*V* (Å^3^)	1523.74 (6)	1577.59 (9)	4473.3 (2)
*Z*	4	4	12
Data collection			
No. of measured, independent and observed [*I* > 2σ(*I*)] reflections	15,809, 2939, 2936	3012, 3012, 2963	31367, 7814, 7731
*R*_int_	0.020	0.024	0.022
(sin θ/λ)_max_ (Å^−1^)	0.617	0.618	0.618
Refinement			
*R*[*F*^2^ > 2σ(*F*^2^)], *wR*(*F*^2^), *S*	0.026, 0.072, 1.06	0.034, 0.092, 1.05	0.033, 0.090, 1.05
No. of reflections	2939	3012	7814
No. of parameters	234	240	547
No. of restraints	2	30	2
Δ_max_, Δ_min_ (eÅ^−3^)	0.19, −0.12	0.22, −0.14	0.28, −0.20
Absolute structure	Flack x determined using 1439 quotients [(I+)-(I−)]/[(I+)+(I−)] [17].	–	Flack x determined using 3275 quotients [(I+)-(I−)]/[(I+)+(I−)] [17].
Absolute structure parameter	0.07 (5)	–	0.09 (8)

**Table 2:** Antimicrobial activity of tested chemicals presented as minimal inhibitory concentrations (MICs) and minimal bactericidal concentrations (MBCs) or minimal fungicidal concentrations (MFCs) against bacteria and yeasts, respectively.

	Chemical	EJMCh-6		EJMCh-9		EJMCh-13		Standard Drug	
Microorganism		mg/L						Vancomycin	
**Gram-positive bacteria**		MIC	MBC	MIC	MBC	MIC	MBC	MIC	MBC
*S. aureus* ATCC 25923		>1000	Nd	31.25	1000	15.6	125	0.98	0.98
*S. aureus* ATCC BAA-1707 *		>1000	Nd	31.25	1000	15.6	1000	0.98	0.98
*S. epidermidis* ATCC 12228		>1000	Nd	7.8	1000	15.6	15.6	0.98	0.98
*M. luteus* ATCC 10240		>1000	Nd	7.8	1000	7.8	62.5	0.12	0.12
*E. faecalis* ATCC 29212		>1000	Nd	15.6	1000	15.6	1000	1.95	3.9
*B. cereus* ATCC 10876		>1000	Nd	15.6	1000	15.6	15.6	0.98	15.6
**Gram-negative bacteria**								Ciprofloxacin	
*S.* typhimurium ATCC 14028		>1000	Nd	>1000	>1000	>1000	Nd	0.061	0.06
*E. coli* ATCC 25922		>1000	Nd	>1000	>1000	>1000	Nd	0.015	0.08
*P. mirabilis* ATCC 12453		>1000	Nd	250	1000	>1000	Nd	0.030	0.03
*K. pneumoniae* ATCC 13883		>1000	Nd	>1000	>1000	>1000	Nd	0.122	0.24
*P. aeruginosa* ATCC 9027		>1000	Nd	>1000	>1000	>1000	Nd	0.488	0.98
**Yeasts**		MIC	MFC	MIC	MFC	MIC	MFC	Nystatin	
*C. albicans* ATCC 102231		>1000	Nd	>1000	>1000	>1000	>1000	0.48	0.48
*C. parapsilosis* ATCC 22019		>1000	Nd	>1000	>1000	>1000	>1000	0.24	0.48
*C. glabrata* ATCC 90030		>1000	Nd	>1000	>1000	500	>1000	0.24	0.48

* Methicillin-resistant Staphylococcus aureus (MRSA); Nd—not detected.

## Data Availability

Data are contained within the article.

## Supplementary Materials

The following supporting information can be downloaded at: https://www.mdpi.com/article/10.3390/ijms24043319/s1.
