# Supplementary material for: Structure and Microbiological Activity of 1H-benzo[d]imidazole Derivatives
Source: Int J Mol Sci. 2023 Feb 7;24(4):3319. doi: 10.3390/ijms24043319 (PMC9961232; doi:10.3390/ijms24043319)
Supplement: Supplementary file 1 [file ijms-24-03319-s001.zip › ijms-2141520-supplementary.pdf]

International Journal of *Molecular Sciences*

MDPI

SUPPLEMENTARY MATERIALS

Structure and Microbiological Activity of 1*H*-benzo[*d*]imidazole Derivatives

Andrzej Olczak [1], Tomasz Pawlak [2,*], Sylwia Kałużyńska [1], Katarzyna Gobis [3], Izabela Korona-Głowniak [4], Katarzyna Suśniak [4], Marcin Zaborowski [5] and Małgorzata Szczesio [1]

[1] Institute of General and Ecological Chemistry, Faculty of Chemistry, Lodz University of Technology, Zeromskiego 116, 90-924 Lodz, Poland
[2] Centre of Molecular and Macromolecular Studies, Polish Academy of Science, Sienkiewicza 112, 90-363 Lodz, Poland
[3] Department of Organic Chemistry, Faculty of Pharmacy, Medical University of Gdańsk, Gen. Hallera Ave. 107, 80-416 Gdańsk, Poland
[4] Department of Pharmaceutical Microbiology, Faculty of Pharmacy, Medical University of Lublin, Chodźki Street 1, 20-093 Lublin, Poland
[5] Dean Office's, Faculty of Chemistry, Lodz University of Technology, Zeromskiego 114, 90-543 Lodz, Poland
* Correspondence: tpawlak@cbmm.lodz.pl

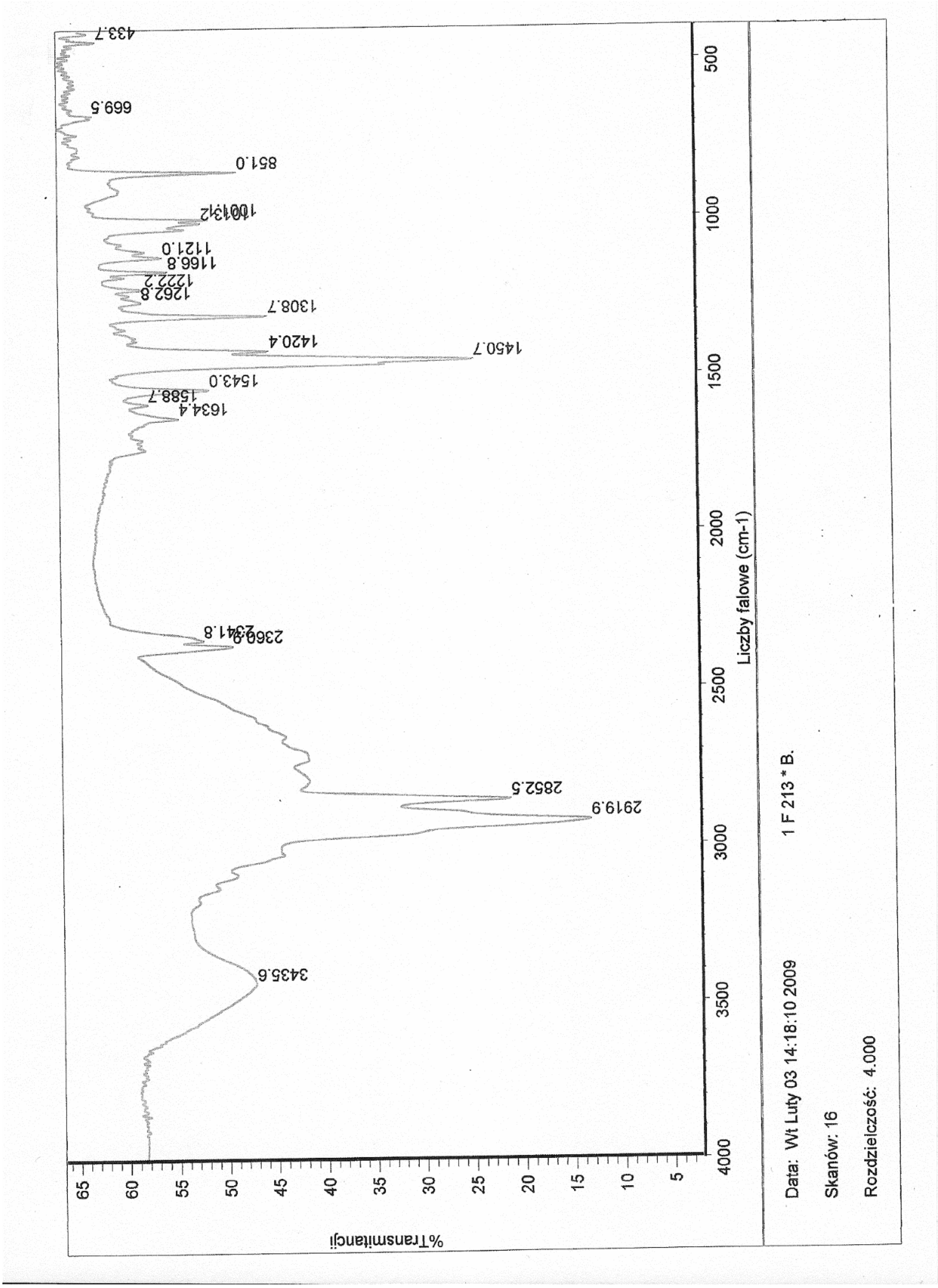

Figure S1. IR spectrum of EJMCh-6.

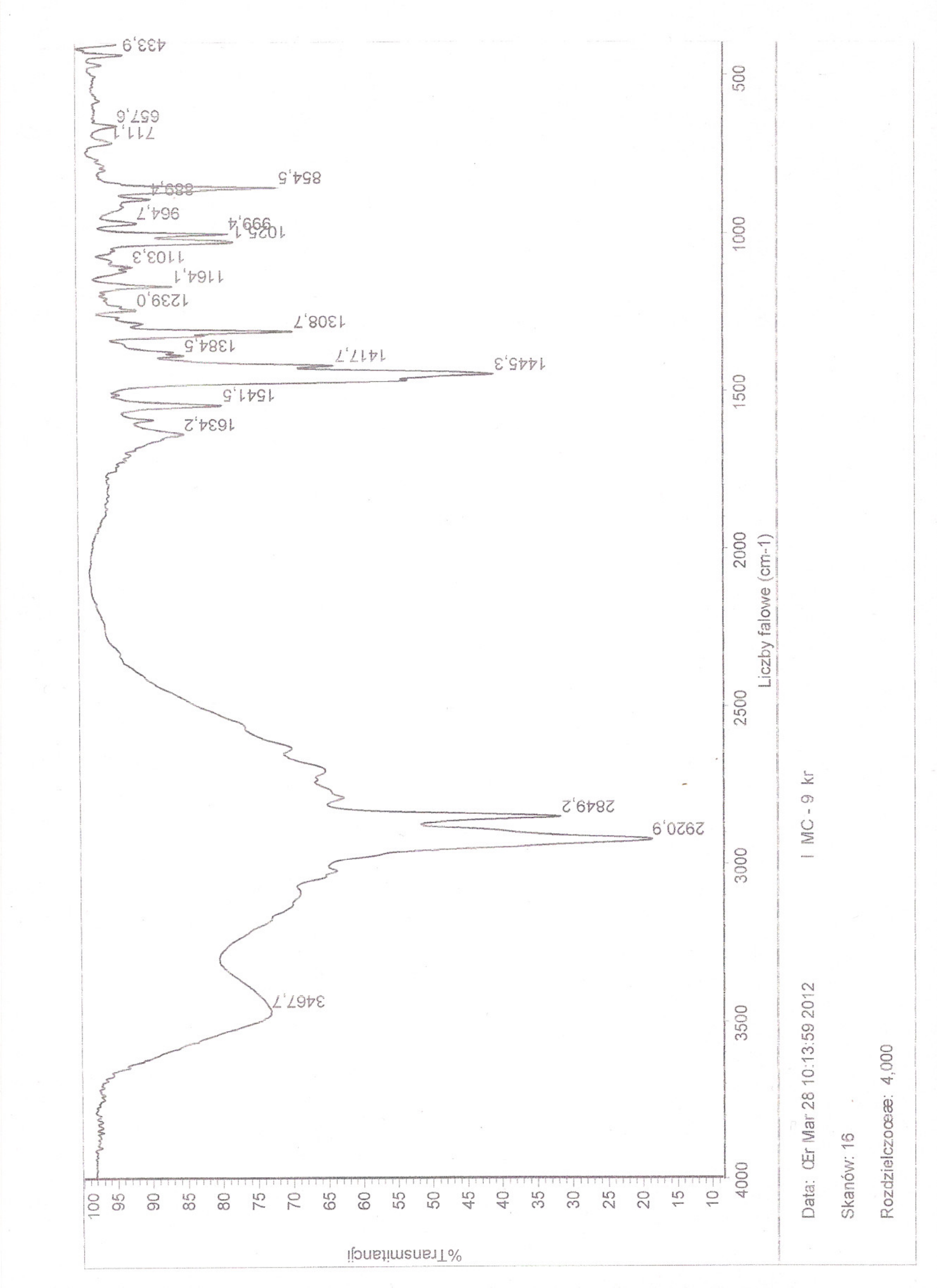

Figure S2. IR spectrum of EJMCh-9.

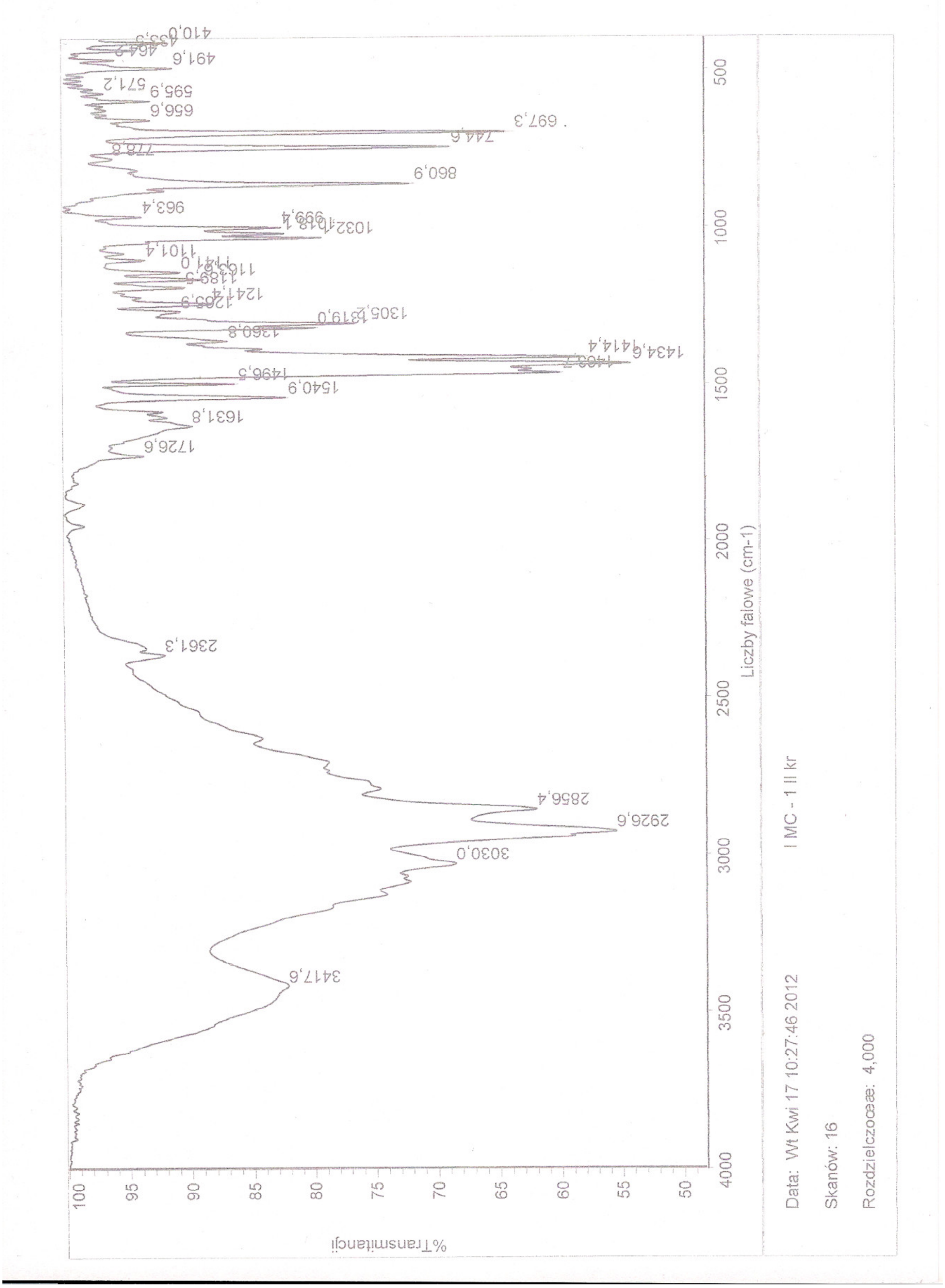

Figure S3. IR spectrum of EJMCh-13.

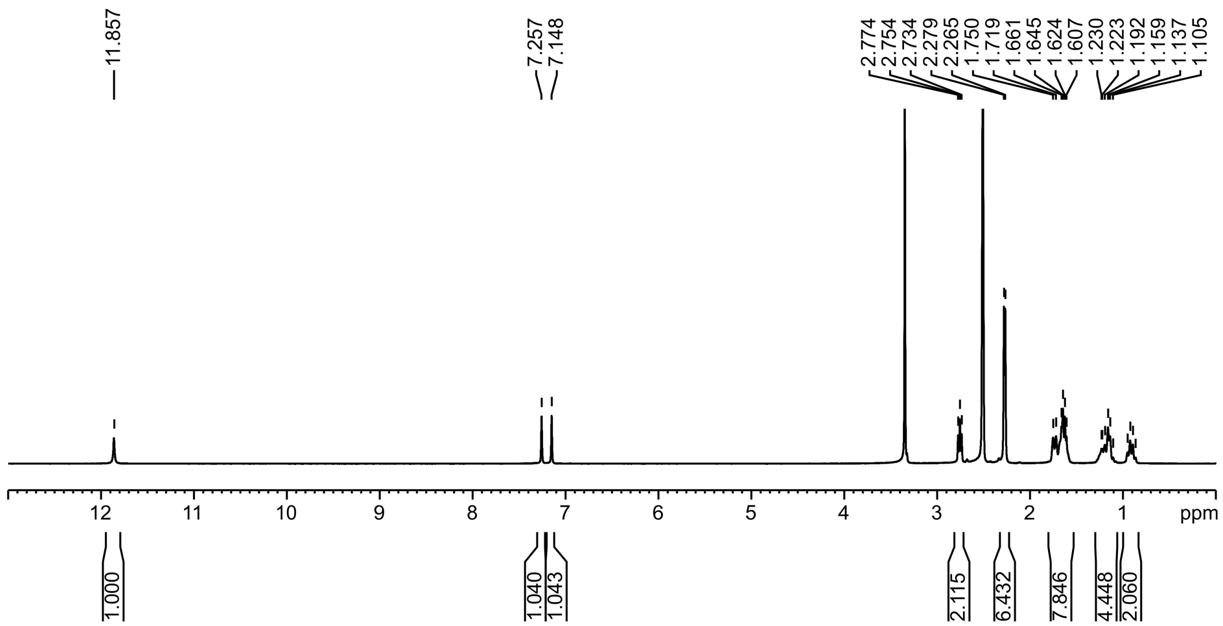

Figure S4. Solution-state ^{1}H NMR (DMSO-d6) of **EJMCh-6**.

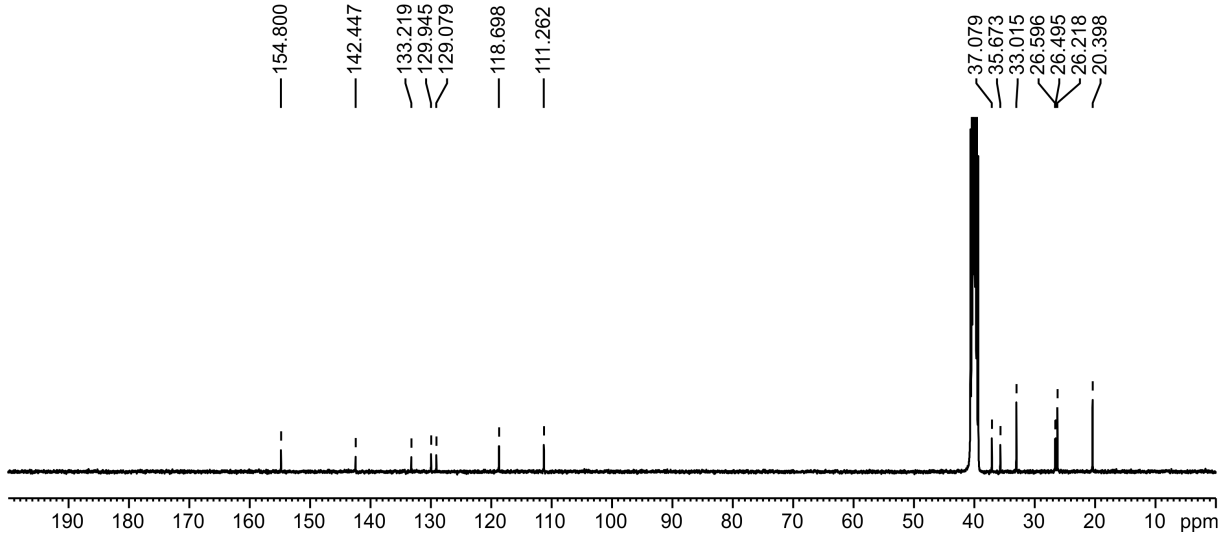

Figure S5. Solution-state ^{13}C NMR (DMSO-d6) of **EJMCh-6**.

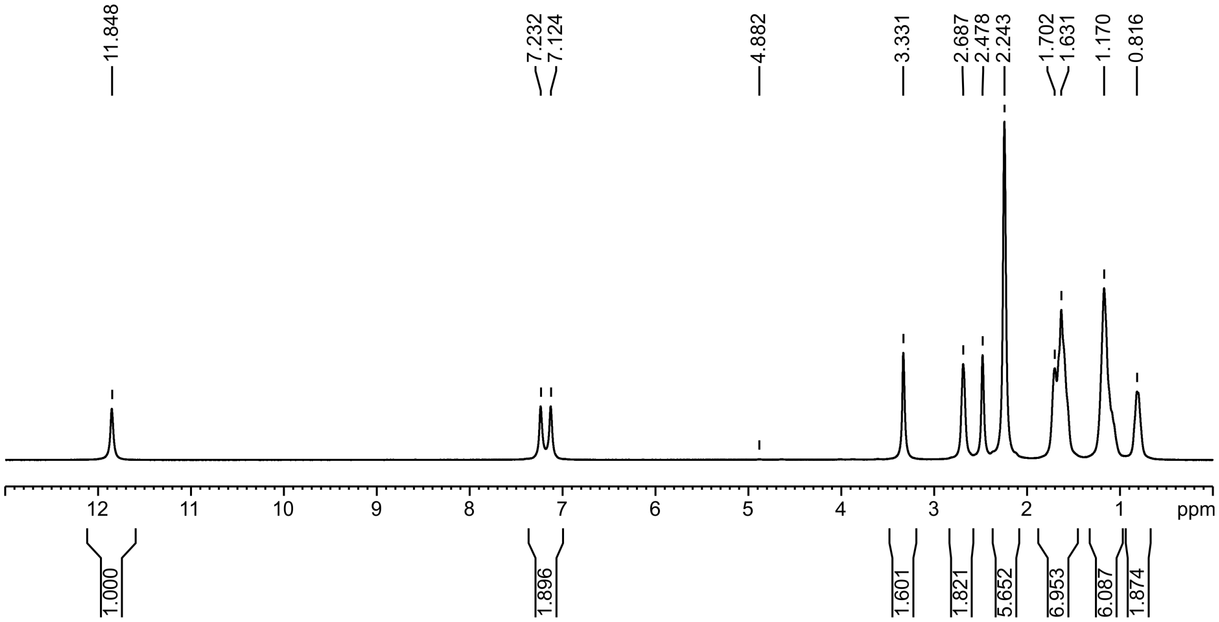

Figure S6. Solution-state ^{1}H NMR (DMSO-d6) of **EJMCh-9**.

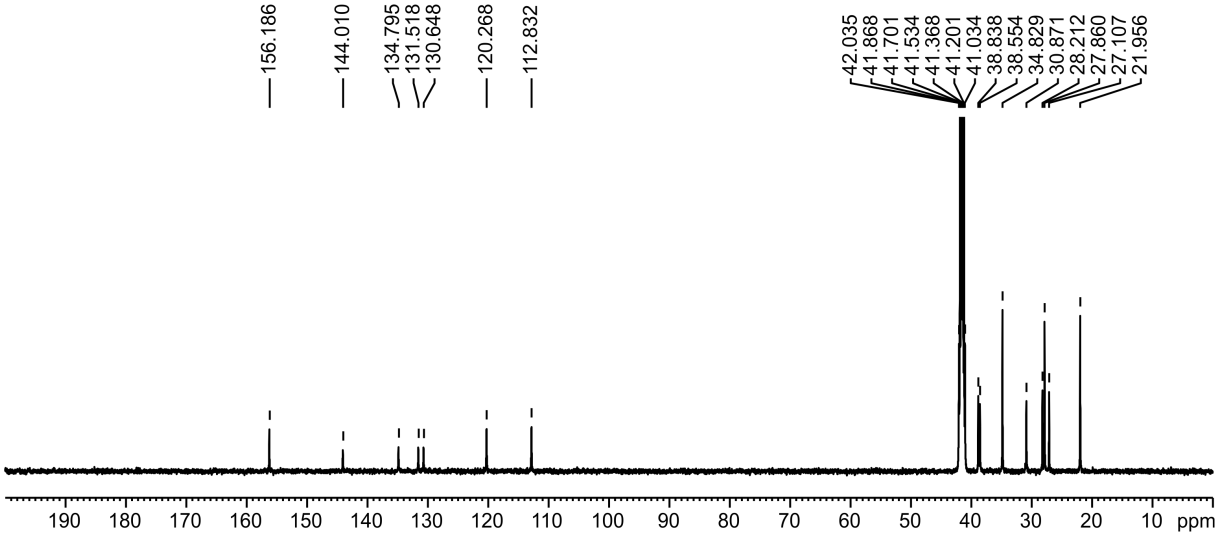

Figure S7. Solution-state ^{13}C NMR (DMSO-d6) of **EJMCh-9**.

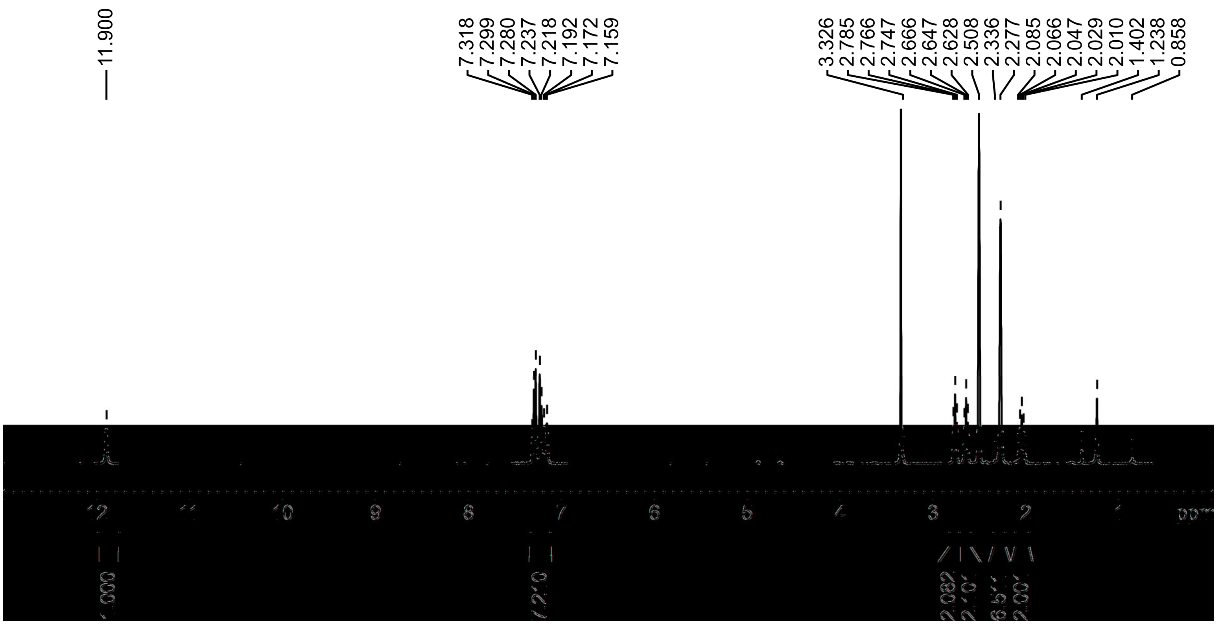

Figure S8. Solution-state ^{1}H NMR (DMSO-d6) of **EJMCh-13**.

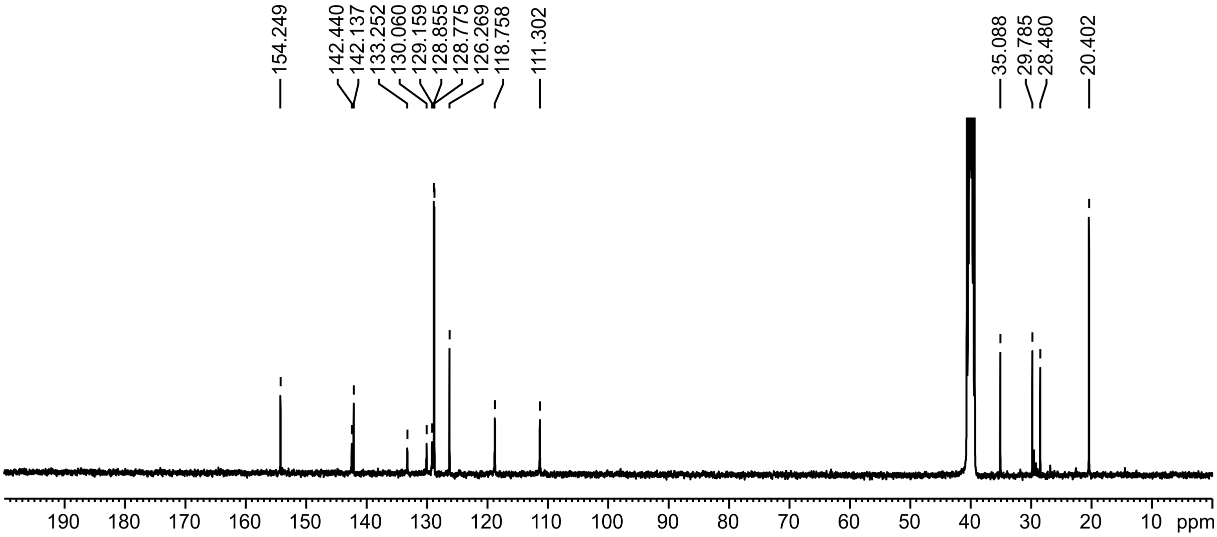

Figure S9. Solution-state ^{13}C NMR (DMSO-d6) of **EJMCh-13**.
